# Gene copy number and function of the APL1 immune factor changed during *Anopheles* evolution

**DOI:** 10.1186/s13071-019-3868-y

**Published:** 2020-01-13

**Authors:** Christian Mitri, Emmanuel Bischoff, Karin Eiglmeier, Inge Holm, Constentin Dieme, Emma Brito-Fravallo, Abbasali Raz, Sedigheh Zakeri, Mahdokht I. K. Nejad, Navid D. Djadid, Kenneth D. Vernick, Michelle M. Riehle

**Affiliations:** 1 Unit of Insect Vector Genetics and Genomics, Department of Parasites and Insect Vectors, Institut Pasteur, Paris, France; 2 CNRS Unit of Evolutionary Genomics, Modeling and Health (UMR2000), Institut Pasteur, Paris, France; 3 Present Address: Wadsworth Center, New York State Department of Health, Slingerlands, NY USA; 4 Malaria and Vector Research Group, Biotechnology Research Center, Institut Pasteur of Iran, Tehran, Iran; 5 Department of Microbiology and Immunology, Medical College of Wisconsin, Milwaukee, WI USA

**Keywords:** Mosquito, Insect immunity, Gene family, Gene essentiality, Gene neofunctionalization

## Abstract

**Background:**

The recent reference genome assembly and annotation of the Asian malaria vector *Anopheles stephensi* detected only one gene encoding the leucine-rich repeat immune factor APL1, while in the *Anopheles gambiae* and sibling *Anopheles coluzzii*, APL1 factors are encoded by a family of three paralogs. The phylogeny and biological function of the unique APL1 gene in *An. stephensi* have not yet been specifically examined.

**Methods:**

The APL1 locus was manually annotated to confirm the computationally predicted single APL1 gene in *An. stephensi*. APL1 evolution within *Anopheles* was explored by phylogenomic analysis. The single or paralogous APL1 genes were silenced in *An. stephensi* and *An. coluzzii*, respectively, followed by mosquito survival analysis, experimental infection with *Plasmodium* and expression analysis.

**Results:**

APL1 is present as a single ancestral gene in most *Anopheles* including *An. stephensi* but has expanded to three paralogs in an African lineage that includes only the *Anopheles gambiae* species complex and *Anopheles christyi*. Silencing of the unique APL1 copy in *An. stephensi* results in significant mosquito mortality. Elevated mortality of APL1-depleted *An. stephensi* is rescued by antibiotic treatment, suggesting that pathology due to bacteria is the cause of mortality, and indicating that the unique APL1 gene is essential for host survival. Successful *Plasmodium* development in *An. stephensi* depends upon APL1 activity for protection from high host mortality due to bacteria. In contrast, silencing of all three APL1 paralogs in *An. coluzzii* does not result in elevated mortality, either with or without *Plasmodium* infection. Expression of the single *An. stephensi* APL1 gene is regulated by both the Imd and Toll immune pathways, while the two signaling pathways regulate different APL1 paralogs in the expanded APL1 locus.

**Conclusions:**

APL1 underwent loss and gain of functions concomitant with expansion from a single ancestral gene to three paralogs in one lineage of African *Anopheles*. We infer that activity of the unique APL1 gene promotes longevity in *An. stephensi* by conferring protection from or tolerance to an effect of bacterial pathology. The evolution of an expanded APL1 gene family could be a factor contributing to the exceptional levels of malaria transmission mediated by human-feeding members of the *An. gambiae* species complex in Africa.
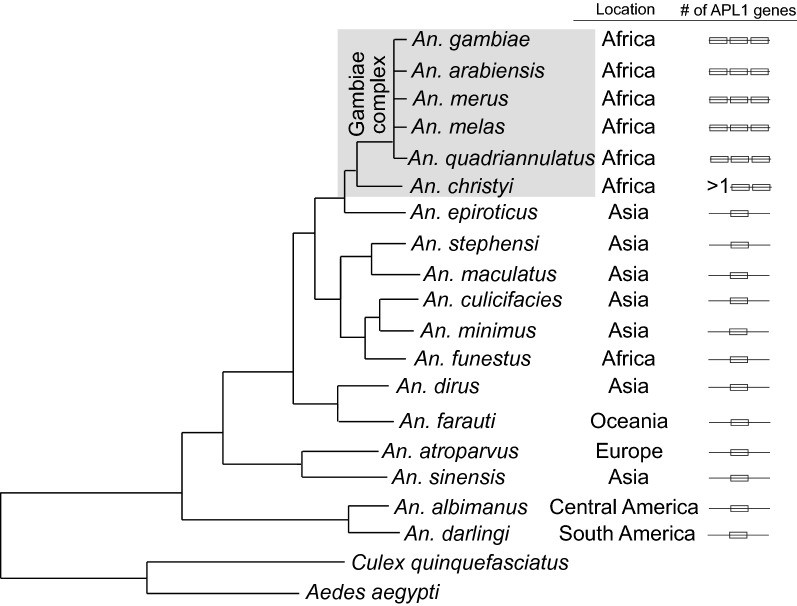

## Background

Malaria remains a serious global public health concern. Human malaria is transmitted by *Anopheles* mosquitoes and among > 450 extant *Anopheles* species, approximately 40 are considered dominant malaria vector species (DVS) [1]. About 90% of global *Plasmodium falciparum* transmission occurs in Africa, where the most important DVS on earth are members of the *Anopheles gambiae* species complex (hereafter, Gambiae complex), including the widespread *Anopheles coluzzii*. An important Asian DVS is *Anopheles stephensi*, which has recently been recognized as an invasive vector species, expanding disease transmission along with its geographical range [2, 3].

The heterogeneity among *Anopheles* species for malaria vectorial capacity can have multiple causes. The first is host-feeding behavior, because animal-feeding species do not have the opportunity to acquire and transmit a human pathogen. Consequently, human-biting preference is the most fundamental prerequisite of malaria vectorial capacity [4, 5]. Among human-feeding DVS, there is apparent variation in vectorial capacity, suggested by large geographical differences in human malaria infection prevalence, with about 90% of global prevalence located in Africa [6]. Some of this global geographical variation could be caused by ecology, if some niches, for example in humid sub-Saharan Africa, are particularly favorable to mosquito abundance and longevity, promoting malaria transmission [7–9]. Finally, vector genetic differences can also underlie physiological difference in vector competence for *P. falciparum* in nature [10–12], but the mechanisms underlying *Anopheles* susceptibility to human malaria in nature are not understood. Several tens of *Anopheles* genes are known from laboratory studies to control malaria infection of the vector, but involvement of these genes in modulating natural transmission has not been confirmed by genetic association in the natural vector population.

The best described mechanism of mosquito immunity in laboratory studies is a ternary immune complex in the Gambiae complex, comprised of the leucine-rich repeat (LRR) proteins APL1 and LRIM1 and the complement-like factor TEP1 [13–15]. APL1 is present in the Gambiae complex as a family of three paralogs, APL1A, APL1B and APL1C [14]. The paralogs display distinct spectra of protection for different pathogen classes [16–18]. APL1A activity inhibits development of the human parasite *P. falciparum*, while APL1C activity inhibits rodent malaria species [14] and APL1B modulates protection against both *P. falciparum* and the rodent parasites [17].

The recent reference genome assembly and annotation of the Asian malaria vector *An. stephensi* revealed only one APL1 gene rather than three paralogs as in the Gambiae complex [19]. Here, we experimentally validate the computationally predicted single APL1 gene in *An. stephensi*. Phylogenomic analysis indicates that a single copy of APL1 represents the ancestral anopheline state, while the expansion to three APL1 paralogs is derived, and among DVS is found only in the African lineage that includes the Gambiae complex. *Anopheles stephensi* APL1 was previously tested for effect on *P. falciparum* [20] and response to kinase signaling [21], but the biological function of the unique APL1 gene has not yet been specifically examined, nor compared to the function of the expanded APL1 locus. We find that the single-copy ancestral APL1 gene and the expanded APL1 locus display distinct functional phenotypes for host survival and protection against *Plasmodium* infection. The expanded APL1 locus is found in the most efficient DVS in the world, the Gambiae complex, which poses the question whether the apparent correlation of APL1 copy number with efficient malaria transmission is accidental or, at least in part, causal.

## Methods

### Mosquitoes

*Anopheles stephensi* SDA-500 strain was initiated in Pakistan [22] and *Anopheles coluzzii* Ngousso strain was initiated in Cameroon [23]. Both strains are housed in the insectaries of the CEPIA platform at the Institut Pasteur, Paris. Mosquitoes were reared under standard conditions at 26 °C and 80% relative humidity, with a 12 h light/dark cycle and continuous access to 10% sucrose solution in cotton pads [17].

*Anopheles stephensi* samples used for APL1 population variation analysis were 6 individuals from a colony initiated at Chabahar, Iran in 2011, 6 individuals from a colony initiated at Bandar-Abbas, Iran in 2008 (both strains maintained at the Institut Pasteur of Iran) and 1 wild-caught individual from Bandar-Abbas. An ~ 800 bp portion of the APL1 coding sequence was amplified from individuals using *An. stephensi* APL1 primers Iran40F and Iran06R. Amplicons of individuals were sequenced, and variant calls were confirmed on both strands by visual examination of ABI sequence chromatogram trace files. *Anopheles coluzzii* APL1 sequences were previously published, generated from the Ngousso colony [24] or wild population [25] and deposited in public archives.

### Phylogenetic analysis of *Anopheles* APL1 gene copy number

The APL1 locus was manually sequenced and sized by PCR in the *Anopheles stephensi* SDA-500 colony housed at Institut Pasteur. Strategy and primers used are indicated in Additional file 1: Figure S1. The annotated *An. stephensi* APL1 genes in the VectorBase genome database [26] are ASTE016290 in the *An. stephensi* SDA-500 strain and ASTEI02571 in the *An. stephensi* Indian strain. The VectorBase assemblies and annotations used, current as of January 2019 were: SDA-500 strain, assembly AsteS1, gene set: AsteS1.7, dated 22 October 2018; and Indian strain, assembly AsteI2, gene set AsteI2.3, dated 21 February 2017.

For phylogenetic analysis of APL1 copy number as presented in Additional file 2: Figure S2, APL1 orthologues for all *Anopheles* species genome assemblies were obtained from VectorBase and sequence was extracted for a window of 60,000 base pairs (bp) centered on the APL1 orthologue(s). Sequences were compared and visualized in a pair-wise fashion using the tBlastX algorithm within the Double Act interface of the Artemis Comparison Tool [27] and visualized using Easyfig [28] to illustrate the number of APL1 family genes across species. Forward and reverse matches were colored the same and percent ID cut-offs were set to a minimum of 50% (light pink in Additional file 2: Figure S2 represents a 50% match and bright red 100% match, areas with less than 50% match are not depicted in color). Each mosquito species was compared directly to the *An. gambiae* PEST genome, the most mature *Anopheles* genome in which the APL1 gene family was originally annotated [14].

For structural comparison of *An. stephensi* APL1 with *An. gambiae* APL1C, peptide sequences were obtained from VectorBase *An. stephensi* assembly SDA-500 and *An. gambiae* assembly AgamP4. Protein motif predictions were carried out and compared using InterPro [29].

### Gene silencing

Double-stranded RNA (dsRNA) specific for target genes was synthesized using the T7 Megascript Kit (Ambion, Waltham MA, USA) as described [16] using indicated primers (Additional file 3: Table S1). For each targeted gene, 500 ng of dsRNA (but not more than 207 nl volume, depending on concentration) was injected into the thorax of cold-anesthetized 1-day-old female mosquitoes using a Nanoject II Auto-Nanoliter Injector (Drummond Scientific, Broomall PA, USA). Mosquitoes were injected with dsRNA specific for the target gene, or with the control dsRNA, dsGFP. The efficiency of gene silencing was monitored 4 days after dsRNA injection in pools of 8 mosquitoes as follows. After total RNA extraction, cDNA synthesis was performed using M-MLV reverse transcriptase and random hexamers (Invitrogen, Carlsbad CA, USA). For each sample, 1µg of total RNA was used in each of three independent cDNA synthesis reactions. Triplicates were pooled and used as template for qPCR analysis. Real-time PCR was performed using an ABI Prism 7900HT sequence detector (Applied Biosystems, Foster City CA, USA). Reactions were prepared in a total volume of 20 μl using SYBR Green PCR master mix (Applied Biosystems, Foster City CA, USA) and 900 nM primers with three serial dilutions of cDNA, each dilution assayed in triplicate. Primers used for verification of gene silencing are indicated (Additional file 3: Table S1). PCR conditions were 95 °C for 10 min followed by 40 cycles of 95 °C for 15 s, 55 °C for 15 s and 60 °C for 45 s. mRNA level was normalized to self (*An. stephensi* or *An. coluzzii*) ribosomal protein rpS7 mRNA in each sample and each gene silencing condition was compared to the control treated with dsGFP.

### *Plasmodium* infection and phenotyping

Mosquitoes were fed on mice infected with *Plasmodium yoelii* strain delta-p230p-GFP [30] at 8–12% parasitemia with mature gametocytes. For parasite development, mosquitoes were maintained at 24 °C and 70% relative humidity on 10% sucrose or 10% sucrose supplemented with penicillin 62.5 µg/ml, streptomycin 100 µg/ml and gentamicin 50 µg/ml. To measure *P. yoelii* infection, mosquito midguts were dissected at day 8 post-infection and oocysts were counted by fluorescence microscopy. Infection phenotypes measured were oocyst infection prevalence, which is the proportion of mosquitoes carrying ≥ 1 oocyst among the total number of dissected mosquitos and oocyst intensity, which is the oocyst count in mosquitoes with ≥ 1 oocyst. Mosquito infection phenotypes were determined for at least two independent biological replicates of ≥ 30 dissected mosquitoes per replicate.

Differences in infection prevalence were statistically tested using the Chi-square test and analysis of oocyst intensity differences used the Wilcoxon signed rank non-parametric test. Statistical differences in prevalence and intensity were first tested independently for each replicate as described above and *P*-values were empirically determined using 100,000 Monte-Carlo permutations. Following independent statistical tests for each replicate and when the direction of change of each independent replicate was concordant, the *P*-values from independent tests of significance were statistically combined using the meta-analytical approach of Fisher [31]. All statistical analyses were carried out using R [32].

### Mosquito mortality curves

Mosquito mortality was monitored in cages of at least 50 mosquitoes, recorded every 2 days until all mosquitoes died. Treatment with dsRNA was performed in 3-day-old mosquitoes and recording of mortality began 4 days after dsRNA injection in 7-day-old mosquitoes. Blood-feeding with or without *P. yoelii* was done 4 days after dsRNA injection in 7-day-old mosquitoes and recording of mortality began 3 days following the normal or infected blood meal in 10-day-old mosquitoes. Beginning at adult emergence, mosquitoes were maintained with 10% sucrose and in the case of antibiotic treatment, supplemented with penicillin 62.5 µg/ml, streptomycin 100 µg/ml and gentamicin 50 µg/ml. Two to three replicates were performed for each condition tested. A Cox proportional hazards regression model was fitted to the data using treatments as predictor terms [33, 34].

## Results

### Phylogeny of APL1 gene expansion from a unique ancestor

Recent *in silico* annotation of the *An. stephensi* reference genome detected a single APL1 gene [19]. This is in contrast to the Gambiae species complex, where APL1 is comprised of a family of three paralogs, APL1A, APL1B and APL1C, with distinct roles in immunity [14, 16]. Because assembly of short-read sequences can be problematic for paralogous families, we first wished to confirm the *in silico* single-gene model for *An. stephensi* APL1. The *An. stephensi* SDA-500 assembly contains an unresolved region with unjoined contigs that could potentially conceal the existence of other APL1 paralogs between APL1 (ASTE016290) and the adjacent gene ASTE008334. We manually sized and sequenced ~ 7 kb of the APL1 locus in SDA-500, which closed the sequence assembly gaps and confirmed the presence of a single APL1 gene in *An. stephensi* SDA-500 (Additional file 1: Figure S1, Additional file 4: Alignment S1).

**Fig. 1:**
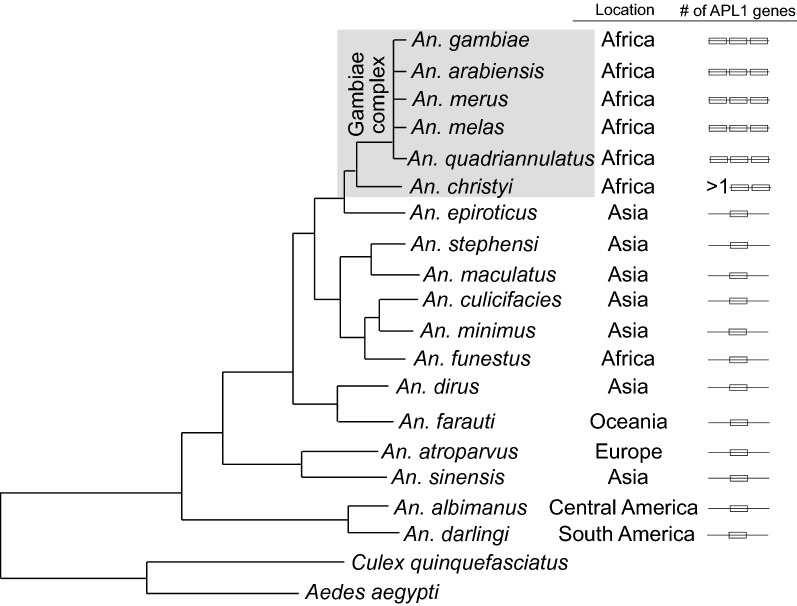
The APL1 gene underwent an expansion in an African *Anopheles* lineage. *Anopheles* phylogenetic tree indicates the number of APL1 gene paralogs present in the genome of 18 *Anopheles* species. Geographical locations of species and the number of APL1 genes in each species are indicated in columns, “Location” and “# of APL1 genes”, respectively. *Anopheles* species worldwide, including *An. funestus* in Africa, carry a single APL1 gene, which is the ancestral state. An exclusively African lineage displays an increased number of APL1 paralogs, including the Gambiae species complex and *An. christyi* (expanded APL1 lineage indicated by shaded box). The five sequenced species of the *An. gambiae* complex clearly carry three APL1 paralogs, while *An. christyi* carries more than one and possibly three, but the genome assembly is poor, thus indicated as > 1 APL1 gene. Phylogeny modified from [35]

We then examined the phylogeny of APL1 in all 19 public reference genomes from 18 *Anopheles* species, which includes two independent assemblies for *An. stephensi* [19, 35]. We accepted genome annotations and did not manually verify the structure of APL1 orthologs in the other genome assemblies as we did above for *An. stephensi* and was previously done for *An. gambiae-An. coluzzii* [14], because we only performed further functional experiments with the latter two species. A single APL1 gene was identified in 12 species, including *An. stephensi*, while the genome assemblies that include the Gambiae complex and *An. christyi* display an expanded APL1 gene family (Fig. 1, Additional file 2: Figure S2). The Gambiae complex members each carry three APL1 paralogs, with the same locus structure as previously described for the sister taxa *An. gambiae* and *An. coluzzii* [14, 25]. The African species *An. christyi*, the closest sequenced relative outside the Gambiae complex, contains at least two APL1 genes and likely a third, but resolution is limited because the *An. christyi* genome assembly is fragmented, with an APL1 locus comprised of three unjoined contigs with intervening sequence gaps (Additional file 2: Figure S2).

The next closest sequenced relative to *An. christyi*, the Asian species *An. epiroticus*, carries a single APL1 gene (Fig. 1, Additional file 2: Figure S2). Based on synteny and the presence in *An. epiroticus* of a homolog of the gene AGAP007034 (located between *An. gambiae* APL1B and APL1C), the single APL1 gene in *An. epiroticus* displays the greatest relatedness to *An. gambiae* APL1C, with APL1B and APL1A presumably arising through duplication events during divergence of the Gambiae complex and *An. christyi* from their common ancestor. The *Anopheles* species carrying an expanded APL1 gene complement do not correspond precisely to the monophyletic Pyretophorus taxonomic group of *Anopheles* species [36, 37]. The Pyretophorus group includes *An. christyi* and the Gambiae complex, which carry an expanded APL1 locus and also *An. epiroticus*, which has only one APL1 gene. Outside the group of *An. christyi* and the Gambiae complex, the evidence clearly supports a unique APL1 gene in all species, although resolution in *An. minimus* is limited due to the poor-quality assembly (Additional file 2: Figure S2). Thus, we conclude that the single APL1 gene found in most sequenced *Anopheles* including *An. stephensi* represents the ancestral state of this locus, while the expansion of APL1 to three genes is a derived state, restricted to the Gambiae complex and *An. christyi*.

Structural comparison of *An. stephensi* APL1 (628 amino acids) with *An. gambiae* APL1C, the Gambiae complex basal paralog (730 amino acids), indicates proteins with 50% amino acid identity and 63% amino acid similarity. Both are members of the “Long” subfamily of leucine-rich repeat immune (LRIM) proteins [38]. Long subfamily LRIMs contain 10 or more leucine-rich repeats. Both *An. stephensi* APL1 and *An. gambiae* APL1C contain predicted secretion signal sequences as well as a coiled-coil domain and a characteristic pattern of cysteine residues represented as C - CC - - C, where the single dash represents 10 amino acids and the double dash represents 30 amino acids. The only notable difference is the absence in *An. stephensi* APL1 of the “PANGGL” domain present in *An. gambiae* APL1C and some alleles of APL1A, a tandemly repeated peptide sequence with unknown function [25].

### APL1 population variation

Genetic polymorphism within the unique APL1 gene in *An. stephensi* was measured by sequencing of individual mosquitoes colonized from the natural population in Iran (Additional file 5: Alignment S2). The unique ancestral APL1 gene in these mosquitoes segregates 7 SNP sites over 1190 bp, or ~ 6 variable nucleotide sites per kilobase (kb). By comparison, the APL1C paralog in the *An. coluzzii* Ngousso colony from Cameroun, measured in the same way, segregates 117 SNP sites in 2924 bp, or ~ 40 variable sites per kb [24], more than six-fold greater polymorphism than the unique *An. stephensi* APL1 gene. *Anopheles stephensi* APL1 is compared to *An. coluzzii* APL1C because APL1C displays the closest orthology to the unique APL1 (Additional file 2: Figure S2). However, in the natural West African population of *An. gambiae* and *An. coluzzii*, paralog APL1A is even more polymorphic than APL1C, displaying approximately double the diversity [25]. The differing levels of diversity of the unique APL1 ancestor and the three APL1 paralogs suggest the genes are exposed to distinct natural selection, likely due to functional differences and points to greater evolutionary constraint upon the single ancestral APL1 gene.

### Depletion of *An. stephensi* APL1 reduces mosquito lifespan

**Fig. 2:**
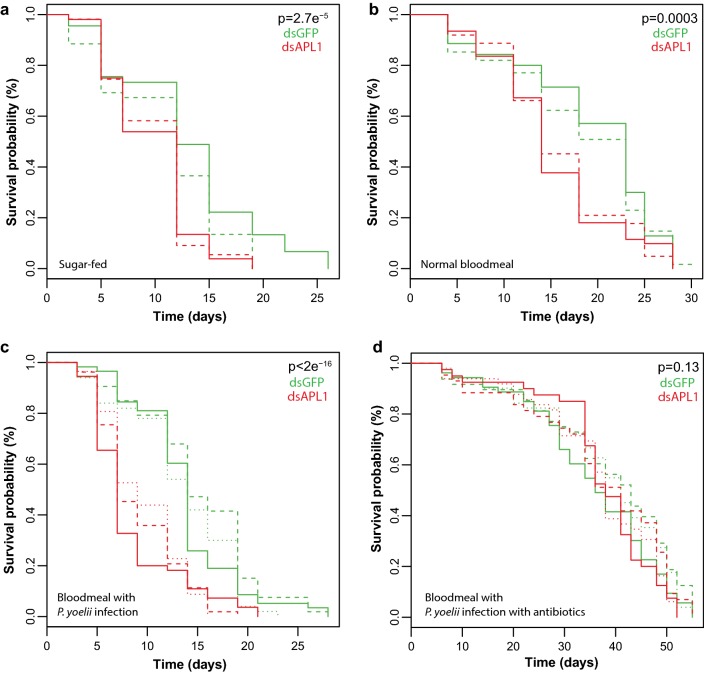
Depletion of APL1 leads to mosquito mortality in *Anopheles stephensi*. Survival curves of *An. stephensi* depleted for APL1 activity by dsAPL1 treatment (red lines) as compared to dsGFP-treated controls (green lines) under different experimental conditions. **a** Sugar-fed mosquitoes. **b** Mosquitoes fed an uninfected normal blood meal. **c** Mosquitoes fed a *Plasmodium yoelii*-infected blood meal. **d** Mosquitoes treated with antibiotics and fed a *P. yoelii*-infected blood meal. Replicate experiments are distinguished by line type (plain, dashed or dotted, respectively). X-axis indicates time after the start of recording. A Cox proportional hazards regression model was fitted to the data using treatment and replicate as predictor terms. The *P*-value associated with the dsRNA treatment term of the Cox model is shown on each panel. Panel **a** Wald statistic = 4.195, *df* = 1, *P* = 2.75e^−5^; Panel **b** Wald statistic = 3.648, *df* = 1, *P* = 0.0003; Panel **c** Wald statistic = 8.376, *df* = 1, *P* < 2e^−16^; Panel **d** Wald statistic = 1.1518, *df* = 1, *P* = 0.129

Depletion of APL1 in *An. stephensi* by RNAi-mediated silencing (silencing efficiency shown in Additional file 6: Figure S3) led to significantly elevated mosquito mortality as compared to mosquitoes treated with a control dsRNA, dsGFP. The effect was seen regardless of whether APL1 depletion was followed by a sugar meal or blood meal (Fig. 2a, b) and the reduction of mosquito lifespan was even more pronounced when APL1 silencing was followed by a *Plasmodium yoelii*-infective blood meal (Fig. 2c). After parasite infection, ~ 70% of APL1-depleted mosquitoes died by day 8 post-infection as compared to ~ 15% mortality in the dsGFP-treated controls.

### Elevated mortality of APL1-depleted *An. stephensi* is rescued by antibiotic treatment

The observed mortality after depletion of an immune gene suggested a potential role in protection from bacterial pathology for APL1 in *An. stephensi*. The three APL1 paralogs found in the Gambiae complex are known to mediate protection from *Plasmodium* infection [17], but their involvement in protection against other pathogens including bacteria has not been reported.

To test the hypothesis that *An. stephensi* APL1 protects from pathogenic bacterial effects, newly emerged adult *An. stephensi* mosquitoes were fed antibiotics in the sugar meal, were then treated with dsAPL1 or dsGFP and were infected with *P. yoelii* parasites. Antibiotic feeding abolished the elevated mortality associated with loss of APL1 function, even in the most pronounced case of *Plasmodium* infection (Fig. 2d). The simplest interpretation is that the activity of APL1 is essential to protect *An. stephensi* from unknown lethal bacterial effects under a range of biological conditions.

### Simultaneous depletion of all three APL1 paralogs does not reduce *An. coluzzii* lifespan

**Fig. 3:**
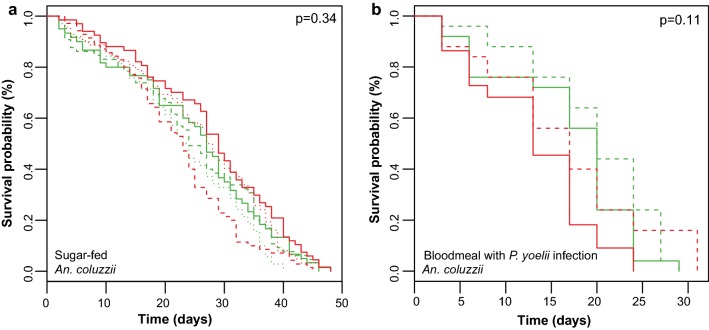
Simultaneous depletion of all three APL1 paralogs in *Anopheles coluzzii* does not cause mosquito morality. **a** Survival curves of *An. coluzzii* depleted for APL1 activity by dsAPL1 treatment (red lines) as compared to dsGFP controls (green lines), for sugar-fed mosquitoes. **b** Survival curves for mosquitoes fed a *Plasmodium yoelii*-infected blood meal. Survival curves from replicates are distinguished by line type (plain, dashed or dotted, respectively). X-axis indicates time after the start of recording, not mosquito age (see Methods). A Cox proportional hazards regression model was fitted to the data using treatment and replicate as predictor terms. The *P*-value associated with the dsRNA treatment term of the Cox model is shown on each panel. Panel **a** Wald statistic 0.95, *df* = 1, *P* = 0.34; Panel **b** Wald statistic = 1.589, *df* = 1, *P* = 0.112

In contrast to the elevated mortality observed in APL1-depleted *An. stephensi*, a mortality effect has not been reported for the APL1 paralogs in *An. gambiae* and *An. coluzzii* [11, 14, 16–18]. To confirm this apparent phenotypic difference between the ancestral and expanded APL1 genes, we tested the effect of loss of all APL1 activity in *An. coluzzii* by depleting all three APL1 paralogs (silencing efficiency shown in Additional file 6: Figure S3). Simultaneous depletion of all three APL1 paralogs did not alter longevity of *An. coluzzii* after sugar feeding (Fig. 3a) nor after *Plasmodium* infection (Fig. 3b). Thus, different from depletion of the single APL1 gene in *An. stephensi*, which caused elevated mortality under these conditions, activity of the three APL1 paralogs in *An. coluzzii* do not display the same function.

### Anti-*Plasmodium* protection by *An. stephensi* APL1 is secondary to protection from elevated mortality

**Fig. 4:**
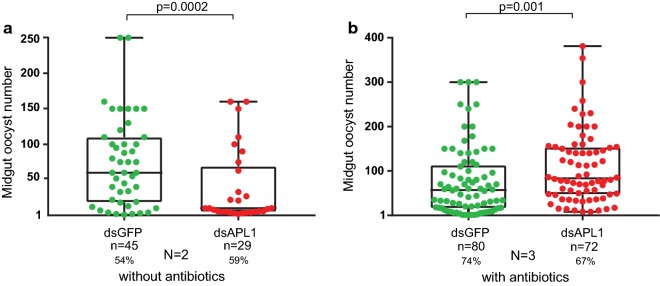
*Anopheles stephensi* APL1 protection from *Plasmodium yoelii* infection is secondary to its antibacterial function. **a** *P. yoelii* oocyst infection intensity in *An. stephensi* mosquitoes treated with dsAPL1 or control dsGFP, both without antibiotic treatment. Oocyst intensity is the oocyst count in mosquitoes with ≥ 1 oocyst, to avoid confounding with infection prevalence. Oocyst infection prevalence, the proportion of mosquitoes carrying ≥ 1 oocyst, is indicated as a percentage below sample sizes. Number of biological replicates is indicated below plots. Combined *P*-value: *χ*^2^ = 22.3529, *df* = 4, *P* = -0.0002 (Replicate 1, W = 30.5, *P* = 0.0075; Replicate 2, W = 226.5, *P* = 0.002). **b** As in **a**, but mosquitoes were subject to antibiotic treatment before *Plasmodium* exposure. Combined P-value, *χ*^2^ = 21.85, *df* = 6, *P* = 0.001 (Replicate 1, W = 1144.5, *P* = 0.009; Replicate 2, W = 463.5, *P* = 0.043; Replicate 3, W = 40, *P* = 0.05549)

Depletion of the unique APL1 gene in *An. stephensi* led to decreased *P. yoelii* parasite load (Fig. 4a). However, the APL1-depleted *An. stephensi* were already compromised due to their elevated mortality and we hypothesized that they may have been physiologically unable to support *Plasmodium* development. In the presence of antibiotics, these mosquitoes carried significantly greater *P. yoelii* infection loads as compared to dsGFP-treated controls (Fig. 4b). Thus, controlling for the mortality effect of APL1 depletion in *An. stephensi* revealed an underlying anti-*Plasmodium* activity of the unique APL1 gene, but the dominant function of APL1 appears to be protection from an elevated mortality phenotype that is complemented by antibiotic provision.

These results are in contrast to silencing of the three APL1 paralogs in *An. coluzzii*, which consistently leads to elevated *Plasmodium* infection levels [16, 17] but not elevated mortality (Fig. 3). Therefore, the three APL1 paralogs confer protection against *Plasmodium* infection independently of the need to protect against mortality from potential bacterial effects. Taken together, these results indicate that the combined phenotype of the three paralogs does not recapitulate the phenotype of the ancestral single gene and that the divergence of the three APL1 paralogs from the unique APL1 ancestor was accompanied by important functional changes. Protection from pathogenic bacterial effects may have been functionally replaced in the expanded-APL1 mosquito lineage by other unknown immune factors or distinct physiological mechanisms.

### APL1 in *An. stephensi* is regulated by both the Toll and Imd signaling pathways

The APL1 paralogs in *An. coluzzii* are transcriptionally regulated by distinct immune signaling pathways. Expression of paralog APL1A is regulated by the transcription factor Rel2, the positive regulator of the Immune deficiency (Imd) immune pathway, while paralog APL1C is regulated by transcription factor Rel1, positive regulator of the Toll pathway [14, 16, 17, 39].

**Fig. 5:**
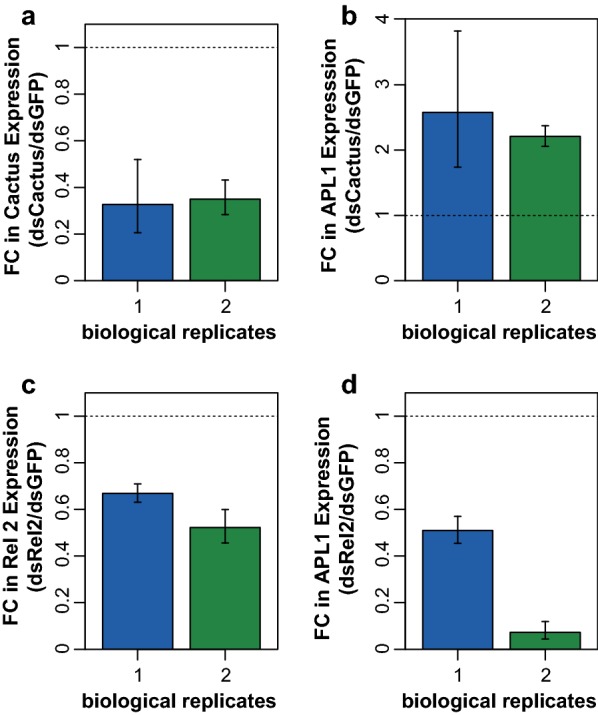
Transcription of *Anopheles stephensi* APL1 is regulated by both Toll and Imd immune signaling pathways. Regulation of expression of the unique *An. stephensi* APL1 gene was queried by silencing the negative regulator of Toll, Cactus (**a** and **b**) or the positive regulator of Imd, Rel2 (**c** and **d**). **a** Cactus expression is efficiently repressed by treatment with dsRNA targeting Cactus (dsCactus). Graph indicates fold change of Cactus expression by dsCactus treatment as compared to dsGFP controls. **b** APL1 expression is augmented by silencing of Cactus, which constitutively activates the Toll pathway. Graph indicates fold change of APL1 gene expression in *An. stephensi* depleted for Cactus by dsCactus treatment, relative to dsGFP treated controls. **c** Rel2 expression is efficiently suppressed by treatment with dsRNA targeting Rel2 (dsRel2). Graph indicates fold change of Rel2 expression by dsRel2 treatment as compared to dsGFP controls. **d** APL1 expression is diminished by silencing of Rel2, which inhibits Imd pathway activity. Graph indicates fold change of APL1 gene expression in *An. stephensi* depleted for Rel2 by dsRel2 treatment, relative to dsGFP treated controls. Transcript abundance is measured by quantitative RT-PCR in two biological replicates as indicated

We tested the effect of these signaling pathways on expression of the unique APL1 gene in *An. stephensi*. Activation of Toll signaling in *An. stephensi* by depletion of the Toll negative regulator, Cactus (Fig. 5a), led to increased APL1 expression (Fig. 5b) and depletion of the Imd positive regulator Rel2 (Fig. 5c) led to reduced APL1 expression (Fig. 5d). Consequently, *An. stephensi* APL1 expression is under control of both the Toll and Imd pathways. A previous study found that overexpression of a Rel2 transgene in *An. stephensi* induced APL1 expression, consistent with our findings, but the response to Rel1 was not tested [20]. Thus, the ancestral unique APL1 gene in *An. stephensi* is regulated by two signaling pathways, Toll and Imd, while after APL1 gene duplication and divergence, these two controlling pathways were subdivided to control of the derived paralogs APL1C and APL1A, respectively.

## Discussion

We find that *An. stephensi* and most other sequenced *Anopheles* species carry a single APL1 gene, which expanded to a family of three paralogs in an exclusively African lineage that includes all members of the Gambiae complex and *An. christyi*. Silencing of the unique ancestral APL1 gene in *An. stephensi* led to elevated mosquito mortality, reversed by antibiotic treatment, which suggested a role for APL1 in protection from pathogenic bacterial effects. The highest mortality was detected in APL1-depleted *An. stephensi* mosquitoes after *Plasmodium* infection, as compared to after a sugar meal or normal blood meal. This result suggests that enteric bacteria could underlie the mortality seen in APL1-depleted *An. stephensi*, because malaria ookinete invasion from the midgut lumen facilitates physical entry of bacteria into epithelial cells and heightens microbial exposure [40].

Thus, we infer that activity of the unique APL1 gene protects *An. stephensi* from effects of the enteric microbiome that are lethal in the absence of APL1. Further work will be required to determine the mechanisms of the bacterial effect and APL1 protection. APL1 could function to modulate bacterial abundance, controlling either specific bacterial taxa or protecting from general dysbiosis; or could mediate tolerance to the stress of bacterial effects such as virulence factors or toxins. APL1 effects on bacteria could potentially influence blood digestion or the peritrophic matrix, although this could not be a primary explanation because elevated mortality after depletion of APL1 is observed with or without a blood meal. Taking into account the present results and the biology of APL1, which is known to be at least a soluble hemolymph factor in *An. gambiae*, we speculate that the unique APL1 in *An. stephensi* may function to protect the hemocoel compartment from enteric bacteria, either as a hemocoel barrier against bacterial escape from the midgut, or as a tolerance factor buffering bacterial pathology.

The function of the unique APL1 gene is distinct from that of the expanded APL1 gene family in *An. coluzzii*, which protects against *Plasmodium* but is not essential for protection from bacterial effects. The unique APL1 gene displays an ancestral immune signaling profile, because its expression is regulated by both Toll and Imd pathways, in distinction to the paralogs in *An. coluzzii*, in which regulation by the immune pathways specialized to different gene family members.

### Function of ancestral and derived APL1 genes

The unique APL1 gene is essential for *An. stephensi* fitness and survival, while the three paralogs combined are not essential for *An. coluzzii* under the same conditions, because their depletion does not have lethal consequences. Gene essentiality is dependent on genomic and biological context, including environmental conditions [41]. The common ancestor of the Gambiae complex-*An. christyi* lineage evolved to exploit an unknown ecological niche, probably in Africa because all species known to carry an expanded APL1 locus are African, and may have encountered new environmental pathogens there [25, 42, 43]. It appears that essentiality of the ancestral unique APL1 gene was lost at the time of the expansion and functional divergence of the three paralogs. The expanded APL1 paralogs evolved new immune roles, exemplified by observed functional differences among the three paralogs in the Gambiae complex [16–18]. However, the paralogs did not simply subdivide the functions of the unique ancestor, because they are not required for protection against bacterial effects under the conditions tested. The expansion of the APL1 gene family was likely accompanied by a suite of other unknown genomic changes necessary for adaptation of the Gambiae complex-*An. christyi* lineage to the new ecological niche, potentially in other immune factors that interact with APL1 such as TEP1 and LRIM1, but this remains to be described. Protection of *Anopheles* from these pathogenic bacterial effects was presumably shifted to other unknown genes or physiological factors, which may have evolved at the same time.

Previous population sequencing revealed that the three APL1 paralogs in the Gambiae complex are exceptionally polymorphic and display signals of adaptive maintenance of variation, including maintenance of alleles that are older than the species of the Gambiae complex [25]. This genetic pattern is consistent with a model of balancing polymorphism maintained by exposure to fluctuating environmental pathogens in a trench warfare dynamic [44]. In contrast, examination of *An. stephensi* APL1 sequences from individual mosquitoes from the Iran population suggests that diversity of the unique APL1 gene is quite low. One potential explanation could be that unique APL1 is under selection mainly to protect the host from relatively stable taxa of enteric bacteria, which could be commensals of the microbiome. Additional population re-sequencing will be required to test these hypotheses.

### APL1 copy number and malaria vectorial capacity

Expanded APL1 copy number does not directly correlate with dominant vector species (DVS) status, but this comparison is confounded with mosquito behavior, because not all expanded-APL1 species are human feeding. The four expanded-APL1 species that are DVS display high human-biting preference (*An. gambiae*, *An. coluzzii*, *An. merus*, *An. melas*), while the other two sequenced species with an expanded APL1 locus, the non-vectors *An. christyi* and *An. quadriannulatus*, are cattle-feeding species in nature [45, 46]. Of these latter two non-vector species, *An. quadriannulatus* is physiologically susceptible to infection with *P. falciparum* when fed on parasitemic blood [46, 47] and permissiveness of *An. christyi* to infection has not been tested.

The more interesting question is whether, among human-feeding species, carriage of the expanded APL1 locus influences the efficiency of malaria transmission. The human-feeding members of the Gambiae complex are considered the most efficient malaria vectors in the world [48, 49] and all of these species carry the expanded APL1 locus. Their efficient malaria transmission could be a secondary consequence of inhabiting African ecological niches that also happen to be particularly favorable to malaria transmission [7, 8, 10]. However, other African vectors such as *An. funestus*, *An. nili*, *An. pharoensis* and *An. moucheti* are DVS, but are often described as locally important secondary vectors and lack the epidemiological impact of the expanded-APL1 Gambiae complex. *Anopheles funestus* carries a single APL1 gene and *An. nili*, *An. pharoensis* and *An. moucheti* have not been sequenced but based on the phylogenetic analysis are also expected to carry the single ancestral APL1 locus.

Thus, the present results raise the question whether the observed correlation of APL1 copy number (and other unknown associated genomic changes) with vectorial efficiency is accidental or biologically meaningful. The ancestral single APL1 protects *An. stephensi* against malaria parasite infection, but this activity is secondary to a dominant and essential function of protection against bacterial effects. Under these conditions, it would not seem adaptive for *Plasmodium* to inhibit the activity of the unique APL1 in order to modulate anti-malaria immunity, because parasite inhibition of unique APL1 immune function in *An. stephensi* would be expected to decrease vector survival and therefore the parasite’s own reproductive fitness. In contrast, in *An. coluzzii* with three APL1 paralogs, malaria immunity and protection from bacterial effects are uncoupled, because loss of APL1 function does not reduce longevity. The separation of anti-*Plasmodium* immunity and protection from bacterial pathology should allow *Plasmodium* (and other pathogens) to subvert APL1-mediated immunity without the risk of provoking host mortality.

## Conclusions

The ancestral and derived APL1 loci, represented by *An. stephensi* and *An. coluzzii*, respectively, display large differences in gene essentiality, function, regulation and genetic diversity. Manipulative experimentation and population genetic analysis will be required to understand the functional and ecological significance of the ancestral and derived APL1 for immunity and malaria transmission in *An. stephensi* and other species.

## Supplementary information

**Additional file 1: Figure S1.** Manual sequence confirmation of unique APL1 gene in *Anopheles stephensi* strain SDA-500. Detailed legend in figure file.

**Additional file 2: Figure S2.** APL1 gene copy number in *Anopheles* genome sequence assemblies. Detailed legend in figure file.

**Additional file 3: Table S1.** Primer sequences.

**Additional file 4: Alignment S1**. FASTA-format sequence data file for manual validation of single APL1 gene in *Anopheles stephensi*. By chromosome synteny, the *Anopheles stephensi* intergenic region between the APL1 gene (ASTE016290 in the SDA-500 assembly) and the adjacent gene (ASTE008334) would contain other APL1 family paralogs, if other APL1 genes exist in *An. stephensi*. The ~ 7 kb intergenic region contains unjoined sequences in the SDA-500 assembly and thus the true size was unknown. The region was amplified and Sanger sequenced by the strategy indicated in Additional file 1: Figure S1 and amplicons were assembled as a single contig, resolving the region between primer ST11 and ST25 (Additional file 3: Table S1). The resolved sequence does not contain sequences related to APL1, confirming the absence of APL1 paralogs in *An. stephensi*.

**Additional file 5: Alignment S2.** FASTA-format sequence data file of amplicon sequences for population variation of APL1 gene in *Anopheles stephensi*. Amplicons of the APL1 gene in *Anopheles stephensi* were amplified from wild mosquitoes and colonies collected in Iran using primers Iran40F and Iran06R and Sanger sequenced.

**Additional file 6: Figure S3.** Efficiency of APL1 gene silencing. Detailed legend in figure file.

## Data Availability

All newly generated sequences are available within the present article as Additional file 4: Alignment S1 and Additional file 5: Alignment S2.

## Abbreviations

bp: base pairs
d: days
dsRNA: double strand RNA
DVS: dominant malaria vector species
RNAi: RNA interference
