# Supplementary material for: Gene copy number and function of the APL1 immune factor changed during Anopheles evolution
Source: Parasit Vectors. 2020 Jan 13;13:18. doi: 10.1186/s13071-019-3868-y (PMC6958605; doi:10.1186/s13071-019-3868-y)
Supplement: Supplementary file 3 — Additional file 3: Table S1. Primer sequences. [file 13071_2019_3868_MOESM3_ESM.docx]

**Additional file 3: Table S1. Primer sequences.** Name preceded by “ST”, primers used for manual sequence annotation of APL1 locus (Additional file 1: Figure S1). Name preceded by “Iran” used to amplify APL1 fragment from the Iran population for resequencing. Sequences of the primers (5’-3’) used for the synthesis of double-stranded RNA for gene silencing (name preceded by “T7”), and for verification of silencing efficiency by quantitative RT-PCR (name preceded by “Ast”). Primers used for *A. coluzzii* are indicated under their primer names.

| ST05 | CCTGCACAGGATACGTATGG |
| --- | --- |
| ST06 | ACTGTCACCGTCACAACTGC |
| ST11 | CTCGGACACTAGACATATCAC |
| ST14 | GCTGGAATACACTTGGAAGTG |
| ST16 | CGAAGGAATGTCACACCGTG |
| ST17 | CGCAGACAGGTGATGATGC |
| ST18 | GCCGAGGCTTCAAAACACC |
| ST19 | TCGGAGTGATTTACTGCTGC |
| ST20 | GGCAGCAGTAAATCACTCCG |
| ST21 | GGAGCTGTCAGATGTCAGG |
| ST25 | GTTCGCAACCGAGTACACC |
| ST28 | ATCGCGCTTGGCAGGTCAC |
| ST29 | AAGGGCAAGCTAATGGAAGC |
| ST30 | AACCAACGCATGATGATGGG |
| ST31 | ACTTTTGACTGGCGATAGCC |
| Iran40F | TCACCCTATCCCACAACGAT |
| Iran06R | ACTGTCACCGTCACAACTGC |
| T7_Ast_ APL1_F | TAATACGACTCACTATAGGatcaaggatccgatgattccg |
| T7_Ast_ APL1_R | TAATACGACTCACTATAGGatgttggattactttattagc |
| T7_Ast_Cactus _F | TAATACGACTCACTATAGGGGAGCTGTGGAATTACGATG |
| T7_Ast_ Cactus_R | TAATACGACTCACTATAGGAGCTGGTACGCCGTCATACC |
| T7_Ast_Rel2 _F | TAATACGACTCACTATAGGCCACGGGACCGATGCCAGAGC |
| T7_Ast_Rel2 _R | TAATACGACTCACTATAGGATGCTGGCCTTGGTCAGCTCC |
| T7_Ast_Stat_1 _F | TAATACGACTCACTATAGGAGCAAGGCGGAACAGTATCTGG |
| T7_Ast_Stat_1 _R | TAATACGACTCACTATAGGCGATTCATCGGTTGTCATATCG |
| Ast_APL1q_V_F | caaaggcccagcatgagcag |
| Ast_APL1q_V_R | tgtgcggaatcatcggatcc |
| Ast_REL2q_F | AGCGCGAGGAGCAACAGATGC |
| Ast_REL2q_R | TTAGCTCTCCGGTAAGTGCAC |
| Ast-Cactus q_F: | GCTCGAGACGACTACTTACG |
| Ast-Cactus q_R | GCTCCACGTTTCGTTAGGTC |
| Ast_ Stat_1q_F | ATCGCGACGGAGCTGCGCGCC |
| Ast_ Stat_1q_R | CGACATGGAGTCAATATTGG |
| Ast_S7q_F | AGGCGATCATCATCTACGTGC |
| Ast_S7q_R | caatgaacacgacgtgctt |
| T7-wAPL1-F  (*A. coluzzii*) | TAATACGACTCACTATAGGATATAACACTAAACAACC |
| T7-wAPL1-R  (*A. coluzzii*) | TAATACGACTCACTATAGGAAAGATCCACGTCAACCA |
| APL1A_VF  (*A. coluzzii*) | GTAAACGAGCTGAGGACTGCGGTGCAGC |
| APL1A_VR  (*A. coluzzii*) | TCTGGTCTTGTATAGTACAATGGAACC |
| APL1B_VF  (*A. coluzzii*) | ACTCGCAAAGCTCAGCAAACAC |
| APL1B_VR  (*A. coluzzii*) | GTGAGAACAAATAAGTTCAAAGTCC |
| APL1C_VF  (*A. coluzzii*) | CTGCTGCAGGGGCTACACGCC |
| APL1C_VR  (*A. coluzzii*) | GGCCCAAGTAACATCATACAC |
